# Pilot Testing a Brief Partner-Inclusive Hybrid Intervention for Perinatal Mood and Anxiety Disorders

**DOI:** 10.3389/fpsyt.2022.735582

**Published:** 2022-05-11

**Authors:** Janelle S. Peifer, Erin Bradley, Gita Taasoobshirazi

**Affiliations:** ^1^Department of Psychology, University of Richmond, Richmond, VA, United States; ^2^Department of Public Health, Agnes Scott College, Decatur, GA, United States; ^3^School of Data Science and Analytics, Kennesaw State University, Kennesaw, GA, United States

**Keywords:** perinatal mood and anxiety disorders, intervention, couples, cognitive behavioral therapy, postpartum depression, postpartum anxiety

## Abstract

The necessity of hybrid and more accessible options for perinatal mood and anxiety disorders (PMADs) has taken on increased urgency in the wake of the COVID-19 pandemic and its lasting impacts. In the New Family Wellness Project (NFWP), participants engage in a hybrid in-person and teletherapy six-session intervention for new parents early in their postpartum period. This small, phase 1 clinical research examined early outcomes of the NFWP's cognitive behavioral intervention on adverse mental health outcomes (i.e., perinatal depression and anxiety, overall mental illness symptoms) and adaptive outcomes and protective factors (i.e., relational health, social support, flourishing, self-efficacy). Despite a small sample size (*N* = 12), paired *t*-tests yielded significant effects for improvements in mental health symptoms at posttest, as well as marginally significant improvements in postpartum anxiety and self-efficacy. Findings suggest the brief, partner-inclusive, hybrid intervention shows promise for further study. Lessons learned from this small phase 1 clinical study and recommendations for revising the intervention prior to future trials are discussed.

## Introduction

Perinatal mood and anxiety disorders (PMADs) persist as the most prevalent perinatal complication, and that contributes to morbidity and mortality for birthing parents (1, 2). A 2017 study on the financial costs associated with untreated PMADs estimated a cost burden of $14 billion for the 2017 birth cohort (3). The COVID-19 pandemic has likely exacerbated this pre-existing concern, with one study citing a 2.5-fold increase in postnatal depression from pre-pandemic levels to April/May 2020 (4). Given the impact of PMADs on such a broad swath of the birthing families and their communities, perinatal mental health advocates have underscored the need for enhanced screening, prevention, and intervention (1).

Various social, physical, and emotional factors interact to influence mental health during the perinatal period in a bidirectional manner. For example, insufficient family support and relational stress can increase the likelihood of PMADs (5) and PMADs can further hinder relational wellness (6). In recent years, perinatal mental health advocates and researchers have heightened their focus on the family system as a whole, incorporating research on fathers and non-birthing parents (7). Research suggests that postpartum depression and anxiety are also common among fathers (8–11) and may be linked to the mother's postpartum depression (9, 12). Because evidence suggests healthy relationships between parents and social support beyond the parental relationship can buffer against negative psychosocial outcomes (13–15), both are potentially important factors when examining PMAD intervention efficacy. Positive relational factors and early mental health care can buffer families from significant negative psychosocial outcomes (15).

While it is likely that couples-based interventions have the potential to equip new parents to navigate psychosocial challenges of parenthood, many interventions employ mother/birthing parent-centered approaches that do not provide consistent monitoring and support for the family unit (16). The historically normative, maternal mental health approach to PMADs may ignore important contextual and social factors that exacerbate symptoms (e.g., isolation) or that could offer relief (e.g., instrumental support, mental health resources, improved relationship functioning). To expand the evidence base, we pilot tested a hybrid in-home and telehealth clinical intervention for new parents that was delivered during the first 6 months postpartum. The intervention combines clinical care (i.e., psychodiagnostic assessment, in-home therapy sessions, support group, between session telehealth enabled clinical consultation and coaching) and support resources (i.e., local perinatal resource guide, crisis support, referral management, and transition planning) to reduce parents' postpartum depression and anxiety. Unlike traditional care models, the units of intervention (couples and individuals), content (combined clinical care and practical support resources), and setting (in-home delivery with telehealth consultation between sessions) can provide a more comprehensive approach and improve new parents' clinical care utilization during the critical early postpartum period.

## Literature Review

The perinatal period can present significant psychosocial challenges. Some parents experience perinatal mood and anxiety disorders (PMADs), which include the onset or worsening of depression and anxiety postpartum or earlier during pregnancy (17). Analyses of CDC's Pregnancy Risk Assessment Monitoring System (PRAMS) 2018 data estimated a 13.2% prevalence of postpartum depression among women in the US, with individual states ranging from 9.7 to 23.5% (18). It is likely that PMAD symptoms are underreported when considering suboptimal screening frequency and quality, as well as mental health stigma (19, 20), particularly for Hispanic and Black women (21). Research suggests that postpartum depression and anxiety also occurs among fathers (8–11) and may be linked to the mother's postpartum depression (9, 12). Perinatal mental health concerns can also include obsessive compulsive disorder for either parent (22, 23) and, in rare cases, psychosis mental health risks for offspring later in life (24–27). In addition, post-traumatic stress disorder after birth, which may occur at a higher rate for non-vaginal deliveries, can contribute to acute disruptions to attachment and nursing processes (28). Early intervention is vital because perinatal mental health issues can increase the risk for harm to one's child or self-harm, including suicide (29, 30).

Studies suggest social support and relational health can contribute to parents' wellbeing (31–33). Healthy relationships between parents may serve as a buffer against negative psychosocial outcomes (13–15). Partners can be a source of several types of social support by meeting emotional needs, helping to correct distorted self-evaluations, sharing parenting responsibilities, or making one aware of and encouraging use of available resources, for instance. Social support beyond the parental relationship can also play a protective role (34, 35). People embedded in parents' broader networks can provide additional emotional, informational, instrumental, or appraisal support that increases a parent's capacity to handle stressors. Family and community resources have also been identified as enabling factors for accessing mental health services (36). Synergy between support from personal relationships and access to and utilization of high-quality resources in one's community could facilitate improvements in PMAD symptoms and life satisfaction. Having “layers” of support may buoy new parents who are navigating parenting challenges for the first time.

Unfortunately, parents may encounter significant barriers accessing the mental health care and resources they need to thrive. Beyond an individual's perceived need for care and/or stigma-related hesitancy seeking care, a health care system's structure and available resources often impede perinatal mental health care access, including long wait times, travel time and distance, inadequate provider mental health training to identify or treat concerns, and costs of care with no or insufficient insurance coverage (36–38). Some populations, such as those from marginalized groups may experience barriers more frequently than their counterparts. Consistent with the body of literature on social determinants of health and equity, a review by Bina (36) showed white women were more likely to seek care for PPD compared to non-white women. Individuals with higher levels of education and those born in the United States (compared to foreign-born parents) were also more likely to seek care. Limited English language skills were also noted as a barrier. Interventions that address ethnicity, culture, as well as social and structural barriers can bolster efforts to improve perinatal mental health because research suggests childbirth experiences differ across cultures and identities (21, 34, 39).

## Theoretical Framework

Cognitive behavioral therapy (CBT) is a widely used therapeutic approach with a robust body of evidence supporting its effectiveness (40). CBT has proven effective for addressing depression, anxiety, and perinatal mood and anxiety disorders (16, 41–48) and has been recommended by the US Preventive Services Task Force for use with perinatal patients (48). As a non-pharmaceutical approach, CBT has added value for perinatal populations considering that certain medications may not be safe or are not preferred during the perinatal period.

For the NFWP, CBT was employed to equip individuals to challenge and change maladaptive thinking patterns, acquire or hone adaptive coping strategies, and improve their utilization of support resources. Cognitive restructuring plays a central role in this process. Individuals learn to recognize maladaptive thoughts, question their accuracy, and reframe the thoughts to make them more productive. Restructuring can help reduce cognitive distortions and anxious and depressive symptoms, enabling individuals to break preservative patterns of negative thinking, stress, low self-esteem, and barriers to adaptive action (49). For example, a parent equipped with cognitive restructuring skills can notice an automatic thought fueled by a maladaptive assumption following a parenting mistake (e.g., “I am doing everything wrong, I never should have become a parent.”), recognize that the thought is distorted (e.g., polarized thinking, catastrophizing), and process the thought in a healthier way (e.g., consider past successes, recognize mistakes as a normal part of life, seek informational or practical support to prepare for similar situations in the future). Cognitive restructuring can also motivate people toward behavioral activation. This complementary technique emphasizes the reciprocity between one's behavior and mood and has strong empirical evidence demonstrating its usefulness (50). Through behavioral activation, an individual engages in activities they find meaningful, which provides positive reinforcements such as pleasure or feelings of accomplishment. Both strategies increase positive reinforcement for the behaviors that reduce depressive symptoms and increase adaptive functioning (e.g., social engagement, physical activity, hygiene, and self-care), leading to decreased symptoms.

## Present Study

The present study examines pilot data obtained prior to larger scale trials. The CBT-based intervention implemented for the New Family Wellness Project pilot study was designed to address three primary outcomes of interest: reduced perinatal anxiety, perinatal depression, and overall mental illness symptomatology. The intervention aimed to equip participants with adaptive strategies (cognitive restructuring and behavioral activation) to promote relational health and resource utilization, resulting in reduced symptoms of perinatal anxiety, perinatal depression, and overall mental illness symptoms. The central aim of the study was to explore preliminary evidence to investigate whether participation in the New Family Wellness Project's CBT intervention can reduce adverse symptoms (i.e., perinatal depression, perinatal anxiety, overall mental illness symptoms) and increase adaptive functioning (i.e., relational health, social support, flourishing, self-efficacy) from pretest to posttest?

## Methods

### Procedures

We designed the New Family Wellness Project (NFWP) collaboratively with the Atlanta Birth Center (ABC)–the only freestanding birth center in Atlanta, Georgia that focuses on low intervention, holistic, wrap-around services for birthing parents and their families. Estimates from 2018 PRAMS data suggest a 13.2% prevalence of postpartum depression in the US (range: 9.7–23.5%), which is comparable to that in Georgia (13.6%), the location of our study. Initial meetings and collaborative goal-setting occurred with key ABC stakeholders in January and February 2019. The team revised the NFWP with feedback from community partners in March 2019, applying for initial funding in April 2019 and completing referral training with the nurse midwife, clinical, and administrative team at ABC in April 2019. Recruitment minimums were met by May 2019 and the NFWP began that month, ending in September 2019.

In order to meet the unique needs of new families, therapy sessions occurred in homes for individual mothers and couples every other week after families brought their babies home, with telehealth consultations between in-home sessions. For the initial pilot, the primary investigator for the study (a licensed clinical psychologist), completed all of the intervention sessions. She consulted with a team of two other clinical psychologists on treatment-related questions or concerns. These in-home therapy sessions utilized CBT skill building focused on cognitive restructuring and behavioral activation to assess families' specific goals and provide psychoeducation on social, physical, and emotional wellness postpartum. The six sessions utilized a semi-structured, manualized approach to ensure consistency. Specifically, while the intervention outlined focus topics, flow, and activities for each session, the clinician was able to hone in on topics and areas that arose organically through the session. The six topics for the sessions include:

#### Session 1: Initial Goal-Setting and Assessment

Analyze key concerns and therapeutic goals, baseline functioning, and set collaborative plans for brief therapy. Complete informed consent and gather history via clinical intake. Introduce CBT techniques, skill-building, and practice homework.

#### Session 2: Grounded Value Activity

Identify individual and family values related to child-rearing and develop a plan for behavioral activation and self-care.

#### Session 3: Shared Life Mapping

Map division of labor, practice cognitive reframing for negative self-talk and communication skills with partners.

#### Session 4: New Parent State of the Union/Time vs. Value

Assess needs across several domains (e.g., physical, social, emotional) and facilitate reflection and communication skills to develop action plans for self-care and utilizing resources to cope with strain of new parenthood.

#### Session 5: Next Steps–Transition Plan

Reflect on skills gathered and set goals for continued work–identify gaps and needs (after session, therapist develops a tailored referral and resource list for each family).

#### Session 6: Transition Session

Complete final transition session to terminate, consolidate therapeutic learning, and check connection with continued resources.

Participants completed the multi-instrument NFWP measure (described in detail in the measures section) prior to beginning the study in May 2019 (baseline) and then again 2 weeks after completing their final session (post). We utilized Qualtrics online survey software to distribute the measure.

### Participants

Twenty individuals (10 partner dyads) were recruited to participate in the study and 12 (6 partner dyads) fully completed the pre-post data collection. We employed snowball, direct referral, and other outreach efforts (i.e., posting flyers in local family activity spaces, reaching out to birth workers with referral information, posting on social media groups for new parents) to recruit participants. Eligible participants were first-time parents, 20–40 years old, and pregnant or within 6 months postpartum with their first child. Participants had to be partnered with the parent of the new child and all participants indicated that they were married or in a committed relationship. All participants were within a 30-min driving radius of the Atlanta Birth Center in downtown Atlanta, Georgia. In the final sample of 12 respondents with complete data, nine identified as White, two identified as Black, and one identified as Latino/a. One did not indicate their race. Seven participants identified as female and five identified as male, with a mean age of 32 (SD = 3.97) and a mean annual income of $84,000 (SD = 33,000).

Our research was conducted in accordance with the principles embodied in the Declaration of Helsinki and in accordance with local statutory requirements. The Institutional Review Board at the first author's institution provided approval for the study to be conducted. All participants were above the age of 18 and gave written informed consent to participate in the study. The data were securely stored to protect the participants' confidentiality.

### Measurements

The NFWP measure included nationally-normed and validated measures in addition to open-ended qualitative evaluation questions developed for the study.

We measured perinatal depression utilizing the 10-item Edinburgh Postnatal Depression Scale (EPDS) (51). The EPDS assesses domains of depression and anxiety during pregnancy and for 1 year following birth using a 4-point Likert scale where higher scores are related to higher levels of depressive symptomatology. While practitioners often use EPDS the produced 0–40 summary scores to screen for risk (e.g., scores of 12/13 indicate possible levels of clinical concern for depressive illness), our analyses looked at scores aligned with the 1–4 Likert scale. Sample questions for the EPDS include, “I have been so unhappy I have had difficulty sleeping” and “I have felt scared and panicky for no good reason,” highlighting anxious, depressive, and suicidal concerns that can characterize the expression of postnatal depressive symptoms. Cronbach's alpha was 0.67 at pretesting and 0.87 at posttesting.

The 31-item Postpartum Anxiety Screening Scale (PASS) (52) examined perinatal anxiety on a 4-point Likert scale where participants respond to how often they experience symptoms associated with perinatal anxiety (0 = not at all, 1 = sometimes, 2 = often, 3 = almost always). The PASS assesses four domains: (1) competence and attachment anxieties; (2) infant safety and welfare anxieties; (3) practical baby care anxieties; and (4) psychosocial adjustment to motherhood. Respondents indicate how often over the past month they have experienced anxious instances such as “worry about the baby/pregnancy” or “wanting things to be perfect.” Cronbach's alpha was 0.91 for pretesting 0.93 for posttesting for the scale.

Overall mental illness symptoms were captured via the Diagnostic and Statistical Manual, fifth edition (DSM-5) cross-cutting assessment tool (53). This screening, 23-item measure uses a 4-point Likert scale indicating how much symptoms have bothered the respondent over the past two-week period (1 = none/not at all, 1 = slight/rare, less than a day or two, 2 = mild/several days, 3 = moderate/more than half the days, 4 = severe/nearly every day). The scale examines a range of criteria relevant to 13 categories of diagnosis present in the DSM such as depression, anger, mania and sleep disturbance, anxiety/panic, somatic symptoms, suicidality, memory and cognition, psychosis, intrusive thoughts and obsessive compulsive disorder, disassociation, relational distress and personality functioning, and substance sue. This broad measure helps identify a breadth of concerns and symptoms captured within the DSM-5. Cronbach's alpha was 0.87 at pretesting and 0.88 at posttesting.

We also explore relational health (for romantic partners) utilizing the Relational Assessment Scale (RAS) (54), a 7-item scale that assesses an individual's satisfaction with their relationship; applicable to any type of romantic couple (i.e., married, dating, engaged, and cohabitating) on a 5-point Likert scale with higher scores related to greater relational satisfaction. Sample questions include: “How well does your partner meet your needs?” and “How good is your relationship compared to most?” Cronbach's alpha was 0.78 at pretesting and 0.87 at post-testing.

The Multidimensional Scale of Perceived Social Support (MSPSS) (55) examines perceived emotional and instrumental support from family, friends, and loved ones. This 12-item measure on a 7-point Likert scale where respondents indicate their agreement with statements (1 = very strongly disagree, 2 = strongly disagree, 3 = mildly disagree, 4 = neutral, 5 = mildly agree, 6 = strongly agree, 7 = very strongly agree). The MSPSS includes questions such as “My family really tries to help me” and “I can talk about my problems with my friends.” Cronbach's alpha was 0.88 at pretesting and 0.85 at post-testing.

The 8-item Flourishing Scale (56) provides a single score representing psychological wellbeing, and assesses self-perceived success in relationships, purpose, optimism, and self-esteem. On a 7-point Likert scale (1 = strongly disagree, 2 = disagree, 3 = slightly disagree, 4 = neither agree nor disagree, 5 = slightly agree, 6 = agree, 7 = strongly agree), participants indicate their agreement with questions such as “I am optimistic about my future” and “I lead a purposeful and meaningful life.” Cronbach's alpha was 0.92 at pretesting and 0.85 at post-testing.

The 10-item General Self Efficacy Scale (GSE) (57) examines participants' self-perceived sense of being able to cope with daily hassles and stressful life events with adaptivity (50). The GSE uses a 4-point Likert scale (1 = not at all true, 2 = hardly true, 3 - moderately true, 4 = exactly true) with higher scores associated with stronger senses of self-efficacy. Sample questions include: “I can always manage to solve difficult problems if I try hard enough” and “I can usually handle whatever comes my way.” Cronbach's alpha was 0.90 at pretesting and 0.92 at post-testing.

## Data Analysis

SAS 9 was used for all statistical analyses. All dependent variables were normally distributed, allowing us to correctly draw conclusions from our statistical test. We conducted paired-samples *t*-tests to evaluate if there were significant differences from pre to post intervention. The data for men and women were combined for analysis because there were no significant differences on any of the mental health variables by gender at either time point (p's > 0.05) and all effect sizes were small to medium. In addition, breaking up the data by gender would further reduce the sample size of the study.

## Result

Table 1 summarizes descriptive statistics for each measure from our sample. Of note, the averages correspond with the Likert scale for each measure as opposed to averages of summed scores for the scales. There was a significant decrease in mental illness symptoms [t(11) = −2.134, *p* = 0.01, Cohen's d = 0.84]. Self-efficacy increased from pre to post (marginally significant), t (11) = −2.03, *p* = 0.06, Cohen's d = 0.59. Differences from pre to post were significant at an alpha level of 0.10 for anxiety [t(11) = 1.91, *p* = 0.08, Cohen's d = 0.55] and flourishing [t(11) = 2.134, *p* = 0.05, Cohen's d =0.62].

**Table 1:** Changes in perinatal mental health measures, new family wellness project, 2019 (*N* = 12).

**Variable**	**Pretest mean (SD)**	**Posttest mean (SD)**	**Effect size Cohen's d**	***p*-value**
Perinatal depression (4-point Likert scale)	1.35 (0.13)	1.34 (0.17)	0.28	0.35
Perinatal anxiety (4-point Likert scale)	0.72 (0.41)	0.57 (0.39)	0.55	0.08^*^
Relational health (5-point Likert scale)	4.40 (0.47)	4.39 (0.34)	0.02	0.95
Emotional and instrumental support (7-point likert scale)	5.86 (0.73)	5.92 (0.67)	0	1.0
Psychological wellbeing (flourishing) (7-point Likert scale)	2.49 (1.00)	2.11 (0.70)	0.62	0.05^*^
Self-efficacy (4-point Likert scale)	3.16 (0.46)	3.27 (0.44)	0.59	0.06^*^
Overall mental illness symptoms (4-point likert scale)	1.60 (0.46)	1.41 (0.38)	0.84	0.01^**^

*The averages are based on response ranges on the Likert scale items for each instrument*.

* *α < 0.10*;

** *α < 0.05*.

## Discussion

Our study aimed to gather early information on participants' experiences with this novel, partner-inclusive, brief cognitive-behavioral intervention for perinatal mental health. Data from the initial pilot will be used to hone and revise future iterations of the New Family Wellness Project (NFWP). The NFWP aligns with the field's increasingly recommended standard of care for postpartum and early pediatric health appointments that bolster early screening and detection of PMADs (58, 59). The format also aligns with findings that show that mothers with PMADs such as postpartum depression prefer group interventions and personal psychotherapy to impersonal based interventions (54).

Early results show some promise for participants' reduction in overall mental illness symptoms and perinatal anxiety. Consistent with our hypothesis, we found a significant reduction in overall mental illness symptoms from pre to post for NFWP participants. The DSM cross-cutting measure captures a range of symptoms including substance use, depression, anxiety, panic, thought disturbance, sleep, memory, intrusions and provides a very cursory insight into symptomatology across a broad spectrum of mental health functioning. Seeing a reduction in these symptoms, even in brief intervention with a small sample size, provides early support worthy of further, more in-depth investigation. The NFWP may have impacted these symptoms given the broad, client-led discussions of multiple intricacies of mental health and wellness. Moreover, the NFWP's incorporation of CBT-skill building for behavioral activation and cognitive restructuring may have bolstered participants' awareness of and skills to engage with mental health concerns. Overall, the sample reported low acuity and often lower levels of mental health symptoms and thus the reduction may also indicate a normal return to baseline functioning as immediate stressors associated with birth become more distal. As new parents' sleep rhythms, access to support and resources, and mastery of parenting skills stabilize, their slightly elevated mental health symptoms may stabilize as well. Future iterations of the study should recruit an ample control group to better understand if the change in functioning is due to time alone or the intervention itself.

Additionally, NFWP participants reported marginally lower levels of anxiety from pre- to post-test. This may relate to the intervention's focus and psychoeducation regarding anxious symptoms and specific CBT skill-building, including thought stopping and mood logging to develop awareness of anxiety cues and skills to address them relationally (e.g., mindfulness). Those who took part in the study also learned communication skills for discussing relational and systemic variables that can impact the expression of anxious traits (e.g., tapping into support systems, communicating needs regarding familial distribution of labor). These skills may have helped reduce anxious symptoms. Again, further investigation would be needed to identify if this reduction was a product of time alone or attributable to the intervention itself.

Additionally, self-efficacy increased somewhat from pre- to post-test. The skill-building and resource-focused aspects of the intervention aimed to help equip new parents with essential skills for wellness and build their confidence at managing the stressors of new parenthood. This focus may have enhanced self-efficacy. Moreover, a therapeutic relationship can help enhance a client's sense of agency and self-efficacy, enhancing their self-awareness and skill set simultaneously. In future studies, focus group data can help us better understand the core aspects of the NFWP or passage of time on self-efficacy.

Contrary to our hypothesis, the NFWP CBT did not enhance all adaptive areas of functioning for participants (excluding self-efficacy which showed a modest rise pre to posttest). In fact, participants showed dips in overall ratings of flourishing from pre- to post-test.

The reduction in flourishing found in pilot results may relate to several considerations and stressors. Generally speaking, new parents report significant dips in satisfaction with life and in their romantic relationships during the adjustment to new parenthood that occurs in the first year postpartum, often called the “parenthood paradox” (60). This dip is most pronounced for first-time parents like the ones included in the study (61, 62). The dimensions captured on the flourishing scale (i.e., psychological wellbeing and self-perceived success in relationships, purpose, optimism, and self-esteem) may face strain with the significant adjustment in identities that accompany the early postpartum period, which is characterized by mental, emotional, and physical stress (60). Additionally, therapeutic work can pinpoint stressors, strains, and misalignments with values that may bring to the surface increased awareness of one's distance from core goals and desires. A brief, 6-week intervention for new parents who are often hindered from participating in external activities and communities that can enhance satisfaction and fulfillment may prove insufficient for addressing flourishing in such a short intervention period. Longitudinal research tracking flourishing over time (e.g., 2 years postpartum) would be helpful to gauge the impact of time, proximity to birth, adjustment to parenthood, and age of child.

Our results did not find pre-post changes in postnatal depression or most of the adaptive functions we hoped to impact (e.g., relational satisfaction, social support, or resource utilization). If not due to our small sample size, these null findings may indicate a need to further enhance modules focusing on these topics in intervention sessions. In particular, the NFWP pilot's short length may insufficiently address depressive symptoms. Future iterations of the NFWP can be built from interventions in primary care and other settings for brief CBT that have demonstrated promising outcomes related to depression (63, 64). In particular, increased focus on goal-setting, identifying patterns of problematic thinking, behavioral activation, mindfulness skills, and additional therapy homework/skills practice may impact depressive symptoms more effectively. Other forms of CBT interventions could also be considered including a hybrid therapy that includes cognitive behavioral and art therapy treatment (CB-ART) (64, 65).

### Limitations

Despite demonstrating some early promise, the NFWP includes several limitations that contextualize our findings and will inform revisions of future iterations of the NFWP. Recruitment of our sample began through a birth center. These settings may differ in important ways from other birth centers or hospitals (e.g., patient populations, geographic location, funding, resources and infrastructure). Moreover, our pilot size was very small with limited representation of people of color and LGBTQIA+ individuals, in particular. Future iterations of our study must prioritize the recruitment and retention of a diverse sample and consider cultural adaptations of the intervention that consider ethnicity, race, religion, and other core aspects of identity. In order to do this, subsequent studies should recruit conscientiously to enhance race, socioeconomic status, gender identity, sexual identity diveristy in particular given their well-established relevance in relation to perinatal mental health. Additionally, larger sample sizes will permit calculation of more stable effect sizes, and sample diversity will permit appropriate subgroup analyses. The six-session duration shows promise, but longer length may allow for more concentrated cognitive behavioral skill-building targeting depressive symptoms. Further, we did not ask the couples for their treatment preference. In the future, allowing couples to select the treatment technique that they believe will work best for them would allow for a more authentic design.

### Implications

Our study may have important implications for perinatal intervention format, particularly the utility of partner-inclusive models of mental healthcare, as well as CBT-based approaches. CBT can empower new parents to identify and respond to maladaptive thoughts and behaviors, which can have an enduring benefit as acquired skills are applied to address a broad range of issues. Although mental health services often are not adequately covered by insurance, if at all, CBT could be less-costly than long-term maintenance of pharmaceutical intervention for perinatal mental health. Moreover, as a non-pharmaceutical alternative to medication, CBT expands parents' access to effective therapy without potential risks to one's child (e.g., exposure to medication via breastmilk).

### Future Directions

Consistent with the aim of phase 1 clinical trials, this pilot study represents a preliminary stage to lay the groundwork for subsequent research for this hybrid, partner-inclusive postpartum mental health intervention. While early findings should be interpreted considering the small sample size, the NFWP demonstrated promising early evidence for its utility in addressing participants' anxious and overall mental illness symptomatology. Future work can leverage more widely-available telehealth to expand access to the intervention in a way that is suitable for a diverse patient population. In addition, it may bolster self-efficacy, increasing participants' sense of agency and ability to manage the unpredictable stressors that characterize the postpartum and parenting periods. Future work will build on these early findings to hone and enhance the New Family Wellness Project in pursuit of a brief, accessible, robust intervention to help meet the diverse mental health needs of new parents and empower families on their journeys toward wellness.

## Data Availability Statement

The raw data supporting the conclusions of this article will be made available by the authors, without undue reservation.

## Ethics Statement

The studies involving human participants were reviewed and approved by Agnes Scott Institutional Review Board. The patients/participants provided their written informed consent to participate in this study.

## Author Contributions

All authors listed have made a substantial, direct, and intellectual contribution to the work and approved it for publication.

## Conflict of Interest

The authors declare that the research was conducted in the absence of any commercial or financial relationships that could be construed as a potential conflict of interest.

## Publisher's Note

All claims expressed in this article are solely those of the authors and do not necessarily represent those of their affiliated organizations, or those of the publisher, the editors and the reviewers. Any product that may be evaluated in this article, or claim that may be made by its manufacturer, is not guaranteed or endorsed by the publisher.
